# Comparative efficacy of botulinum toxin and surgical treatment for acute acquired concomitant esotropia: a systematic review and meta-analysis

**DOI:** 10.3389/fphar.2026.1775497

**Published:** 2026-06-17

**Authors:** Yushan Shi, Kailai Nie, Min Tian, Hongbin Lv, Junting Huang

**Affiliations:** 1 Department of Ophthalmology, The Affiliated Hospital of Southwest Medical University, Luzhou, China; 2 Department of Ophthalmology, West China Hospital, Sichuan University, Chengdu, China; 3 Department of Ophthalmology, Sichuan Provincial People’s Hospital, School of Medicine, University of Electronic Science and Technology of China, Chengdu, China

**Keywords:** acute acquired concomitant esotropia, botulinum toxin, efficacy, meta-analysis, surgery

## Abstract

**Objectives:**

This meta-analysis aimed to compare the efficacy of botulinum toxin (BTX) injection versus surgery for acute acquired concomitant esotropia (AACE).

**Methods:**

PubMed, Embase, Web of Science, China Biomedical Sinomed, and the Cochrane Library were systematically searched for studies comparing BTX and surgery in AACE. The primary outcome was motor success rate, and secondary outcomes included stereopsis recovery and incidence of permanent exotropia. Random-effects models were applied. Sensitivity analyses, subgroup analyses, meta-regression, and publication bias assessment were performed.

**Results:**

Fourteen studies involving 1027 patients were included. At 6 months, motor success rates did not differ significantly between BTX and surgery (OR 0.45, 95% CI: 0.20–1.03, P = 0.06); however, this result was sensitive to the inclusion of a single study, and exclusion of this study favored surgery (OR 0.36, 95% CI: 0.17–0.77, P = 0.008). Subgroup analyses showed that comparable outcomes were observed between BTX and surgery in pediatric patients (OR 1.14, 95% CI: 0.45–2.89, P = 0.78) and in studies using bilateral medial rectus injection (OR 0.98, 95%CI: 0.35–2.70, P = 0.97), with stable sensitivity analyses in both subgroups. Stereopsis recovery at 6 months was similar between the two treatments (near: OR 0.61, 95% CI: 0.32–1.19, P = 0.15; distance: OR 1.05, 95%CI: 0.42–2.61, P = 0.92).

**Conclusion:**

The pooled short-term motor outcome showed substantial heterogeneity and sensitivity to individual studies. More consistent findings were observed in pediatric populations and in studies using bilateral medial rectus injections, where BTX may achieve short-term outcomes comparable to surgery. Stereopsis recovery at 6 months was also similar between the two treatments. However, given the limited number of available studies and the predominance of retrospective designs, adequately powered RCTs and prospective studies are needed to establish more definitive conclusions.

**Systematic Review Registration:**

Identifier: CRD420245127395.

## 1 Introduction

Acute acquired concomitant esotropia (AACE) is defined as the sudden onset of comitant esotropia, typically manifesting with horizontal diplopia and a consistent deviation across all directions of gaze (Wen et al., 2025; Zhang et al., 2025). It predominantly affects older children and adults with otherwise normal ocular motor function and is generally associated with a favorable prognosis for binocular vision recovery (Lekskul et al., 2021). Notably, the global incidence of AACE has risen in recent years, drawing increasing clinical and research attention (Chen et al., 2025; Hayashi et al., 2022; Ui et al., 2026). Surgical correction remains the standard treatment for restoring satisfactory ocular alignment, whereas the invasiveness and surgical related risks underscore the need for effective alternatives. Botulinum toxin (BTX) is a large molecular protein that selectively inhibits acetylcholine release, leading to reduced medial rectus contractility and helping to restore the ocular alignment (Tugcu et al., 2025). Compared with surgery, BTX injection is less invasive, requires shorter anesthesia exposure, and preserves muscular integrity. These characteristics position BTX a potential option for patients seeking to avoid surgery or those with elevated surgical risks.

Recent studies have increasingly compared BTX injections with surgical treatment for AACE. While several studies found that BTX achieved efficacy comparable to surgery (Nguyen et al., 2025; Yu et al., 2024; Lang et al., 2019; Shi et al., 2021), others reported higher success rates with surgery (Liu et al., 2025; Cheung et al., 2024; Suwannaraj et al., 2023). Collectively, these disparate results highlight uncertainty regarding the relative efficacy of BTX and surgery. Therefore, this study aims to systematically synthesize evidence from high-quality clinical studies and perform a comprehensive meta-analysis to evaluate the efficacy and safety of BTX injection versus surgical treatment for AACE.

## 2 Methods

### 2.1 Search strategy

The systematic review protocol was registered with PROSPERO (CRD420245127395) prior to commencement, and the PRISMA checklist is provided in Supplementary Table S1. We systematically searched Embase, PubMed, Web of Science, China Biomedical Sinomed, and the Cochrane Library from their inception until August 6, 2025, without any restrictions on publication date. The search was last updated on 1 March 2026. Search terms included “acute acquired concomitant esotropia,” “acute concomitant esotropia,” “botulinum toxin,” “botulinum Neurotoxin,” “surgery,” “strabismus surgery,” “extraocular muscle surgery,” and “rectus recession.” The full search strategies for all databases are provided in Supplementary Table S2. We also manually screened the reference lists of all included articles and relevant review articles to identify additional eligible studies.

### 2.2 Inclusion and exclusion criteria

Studies were included if they met the following criteria.

1. Populations: patients clinically diagnosed with AACE without evidence of underlying neurological, psychiatric, or other systemic diseases;
2. Interventions and comparators: studies directly comparing BTX injection with conventional surgery as primary treatment for AACE, with a minimum of 6 months of postoperative follow-up;
3. Outcomes: reporting motor success rate as the primary outcome. Motor success was defined as postprocedure deviation within 10 prism diopters (PD) of orthotropia. Studies reporting additional outcomes such as stereopsis recovery rate or incidence of permanent exotropia were also included when available. Stereopsis recovery was defined as the achievement of any detectable stereopsis at the 6-month follow-up that was absent preoperatively;
4. Study designs: randomized controlled trials (RCTs), prospective cohort studies, and retrospective comparative studies;

Studies were excluded if they met any of the following criteria.

1. studies not evaluating BTX for the treatment of AACE, single-arm studies without a comparator, or studies lacking a surgical comparison group;
2. non-peer-reviewed publications (e.g., conference abstracts, preprints, grey literature), letters, commentaries, case reports, reviews, or meta-analyses;
3. studies lacking extractable or calculable outcome data;
4. patients with a history of prior BTX injection or strabismus surgery;
5. studies not published in English or Chinese.

### 2.3 Data extraction and quality evaluation

For each eligible study, data were independently extracted by two investigators (SYS and KLN), and any discrepancies resolved through consultation with a third researcher (JTH). Extracted data included the first author, year of publication, study design, age, AACE duration, follow-up duration, outcome measures, angle of deviation, sample size, number of successful cases in each group, BTX dosage, and BTX injection method (Table 1). All analyses were restricted to participants who completed follow-up, and statistical evaluations were conducted on data from single BTX injections. Risk of bias for each included study was independently assessed by two authors (SYS and KLN) using a modified version of the Newcastle–Ottawa scale (NOS) (Lo et al., 2014). The NOS evaluates three domains: selection (0–4 points), comparability (0–2 points), and outcome assessment (0–3 points). The maximum score is 9, with studies classified as high quality (7–9), moderate quality (4–6), or low quality (≤3).

**TABLE 1:** The characteristics of included studies.

Study	Study design and follow-up	Country	Age, mean/Range, y	Duration of AACE	Angle range (PD)		Sample size, N, BTX/Surgery	Definition of motor success	BTX injection dose	BTX injection method	NOS scores
					Near	Distance					
Yu et al. (2024)	Retrospective 6m, 12m, 24 m	China	BTX: 29.8 Surgery: 29.35	BTX: 1.53 ± 2.01 y Surgery: 4.34 ± 2.84 y	BTX: 24.51 ± 13.31 Surgery: 28.39 ± 13.75	BTX: 25.86 ± 13.38 Surgery: 35.16 ± 13.69	73/31	≤5PD	3.5IU:16–20PD 4IU:21–40PD 4.5IU:40–50PD	M2	8
Nguyen et al. (2025)	Retrospective 6 m,36 m	USA	Overall: 2–10	BTX: 2.5 (0.5–18.0) m Surgery: 7.0 (2.0–13.0) m	NR	BTX: 35 (10–55) Surgery: 35 (12–55)	44/32	≤10PD	5 (3.75–5) IU	M2	8
Liu et al. (2025)	Prospective 1 m,3 m,6 m	China	BTX: 8–54 Surgery: 6–56	NR	BTX: 30 (20–40) Surgery: 30 (20–40)	BTX: 30 (20–40) Surgery: 30 (20–40)	33/27	≤10PD	2.5IU:<30PD 5IU:>30PD	M2	8
Cheung et al. (2024)	Retrospective 6 m,12 m,24 m	USA	Overall: 2–17	BTX: 4.43 (2.2,6.1) m Surgery: 4.93 (2.87,7.1) m	BTX: 35 (30, 45) Surgery: 35 (30, 45)	BTX: 35 (25, 40) Surgery: 35 (30, 40)	47/44	≤10PD	5 (2.5–7.5) IU	M2	7
Suwannaraj et al. (2023)	Retrospective 1 m,3 m,6 m	Thailand	Overall: 5–59	NR	NR	BTX: 30.7 ± 11 Surgery: 30.0 ± 8.5	7/25	<10PD	5IU	M1	8
Suwannaraj et al. (2023)	Retrospective 1 m,3 m,6 m	Thailand	Overall: 5–59	NR	NR	BTX: 56.5 ± 9.4 Surgery: 60.3 ± 12.7	27/55	<10PD	5IU	M1	8
Li et al. (2023)	Retrospective 2w,6 m	China	Overall: 6–50	Overall: 1 (0.08–10) y	BTX: 18 (12.5–25) Surgery: 35 (25–40)	BTX: 20 (15–30) Surgery: 40 (25–45)	51/41	<8PD	3IU:10–25PD 3.5IU:26–35PD 4IU:>35PD	M1	7
Shi et al. (2021)	Prospective >6 m	China	BTX: 22.4 Surgery: 18.0	BTX: 9.60 ± 10.2 m Surgery: 16.5 ± 13.5 m	BTX:27.1 ± 13.8 Surgery: 39.6 ± 11.0	BTX: 32.3 ± 15.4 Surgery: 44.0 ± 11.4	40/20	<10PD	4IU: <30PD 5IU:30–40PD 6IU:40–50PD 7IU:>60PD	M2	7
Lang et al. (2019)	Retrospective 1 day,1 m,6 m	China	BTX: 3–24 Surgery: 3–32	BTX: 3.38 ± 1.7 m Surgery: 2.31 ± 1.35 m	BTX: 41.92 ± 27.65 Surgery: 32.66 ± 24.11	BTX: 47.30 ± 22.69 Surgery: 39.66 ± 20.13	13/16	≤10PD	2.5IU	M3	8
Wan et al. (2017)	Retrospective 6 m,18 m	USA	Overall: 2–10	BTX: 3.0 (0.5–12.7) m Surgery: 6.8 (2.1–12.7) m	NR	BTX: 35 (10–55) Surgery: 35 (12–55)	16/33	≤10PD	5IU	M2	8
Zhang et al. (2024)	Retrospective 1 m,3 m,6 m	China	2.5IU BTX: 4–6 5.0IU BTX: 3–8.5 Surgery: 4–9	2.5IU BTX: 0.29 (0.18–0.47)y 5IU BTX: 0.33 (0.20–0.50)y Surgery: 0.75 (0.5–2.0) y	2.5 IU BTX: 61.25 (59.38–70.63) 5.0 IU BTX: 67.50 (60.00–70.00) Surgery: 61.25 (55.63–70.00)		43/17	<10PD	2.5/5IU	M5	7
Zhang et al. (2023)	Retrospective 1w,1 m,3 m,6 m	China	Overall: 4–49	BTX: <6 m Surgery: ≥6 m	BTX: 36.89 ± 13.79 Surgery: 37.94 ± 19.58		9/24	≤10PD	2IU:<20PD 3IU:20–35PD 4IU:35–50PD 5IU:>50PD	M3	8
Qin et al. (2024)	Retrospective 1m, 3m, 6 m	China	Overall: 6–14	BTX: <6 m Surgery: ≥6 m	BTX: 36.72 ± 12.67 Surgery: 37.24 ± 13.85		34/25	<10PD	4–5IU	M5	8
Wang et al. (2025)	Retrospective 6 m	China	Overall: 3–18	BTX: 4.18 ± 5.05 m Surgery: 9.57 ± 7.38 m	BTX: 41.35 ± 16.90 Surgery:49.2 ± 18.25	BTX: 39.71 ± 14.94 Surgery: 47.0 ± 18.53	17/23	<5PD	2.5IU:<50PD 5IU:>50PD	M2	8
Qi et al. (2023)	Prospective 1 m,12 m	China	BTX: 31.53 Surgery: 32.64	BTX: 12.64 ± 3.46 y Surgery: 12.38 ± 3.76 y	BTX: 26.91 ± 5.74 Surgery: 27.35 ± 5.79		79/81	<10PD	1.25–2.5IU:<20PD 2.5–3.5IU:20–40PD 2.5–5IU:>40PD	M3	8

AACE, acute acquired concomitant esotropia; BTX, botulinum toxin; M1, Uilateral medial rectus muscle injection without conjunctival incision; M2, Bilateral medial rectus muscle injection without conjunctival incision; M3, Unilateral medial rectus muscles with a conjunctival incision; M4, Bilateral medial rectus muscles with a conjunctival incision; M5, Unilateral or bilateral medial rectus muscles with a conjunctival incision; w, week; y, year; m, month; IU, International Unit; PD, Prism degree; NR, not reported.

### 2.4 Statistical analysis

Summary odds ratios (ORs) with 95% confidence intervals (CIs) were calculated by a random effects model with the Mantel-Haenszel method to evaluate the effectiveness of BTX versus surgery for AACE. For outcomes with sparse data and studies containing zero events in one or both arms, the Peto odds ratio method was applied. The primary outcome was treatment success rate, and secondary outcomes included stereopsis recovery and incidence of permanent exotropia. Heterogeneity was assessed using the chi-square (X^2^) test and the I^2^ statistic, and was considered substantial when I^2^ > 50% and P < 0.1. To verify the robustness of the pooled estimates, sensitivity analyses were conducted by sequentially omitting individual studies. Preplanned subgroup analyses were conducted based on factors potentially influencing BTX efficacy, including participant age, preoperative deviation, AACE duration, and injection method, followed by meta-regression analyses to further investigate the sources of heterogeneity. Publication bias was assessed using Begg’s funnel plot and Egger’s test. Data synthesis was conducted using Review Manager version 5.4.1, and publication bias and meta-regression analyses were performed with STATA version 14.0 (StataCorp, College Station, TX, USA). A two-tailed P < 0.05 was considered statistically significant.

## 3 Results

### 3.1 Characteristics of the studies

**FIGURE 1:**
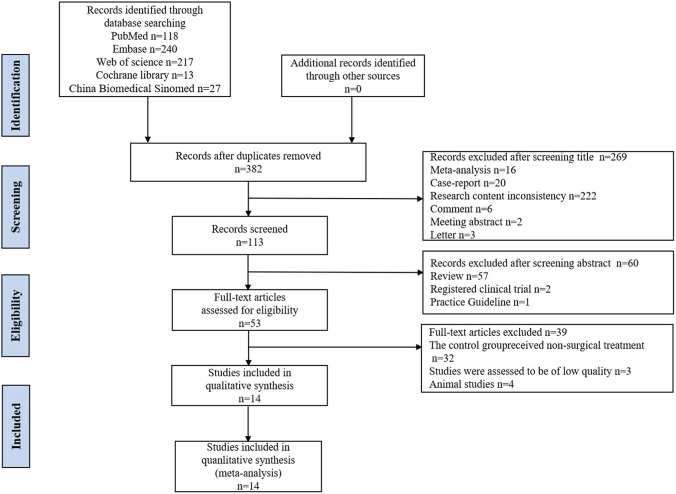
PRISMA flowchart for literature screening.

A total of 615 records were identified through database searches: 118 from PubMed, 240 from Embase, 217 from Web of Science, 13 from the Cochrane Library, and 27 from SinoMed. After removal of 233 duplicates, 382 records were screened for eligibility. Following the assessment of titles, abstracts, and full texts, 14 eligible studies were identified for data analysis (Figure 1). Of these, thirteen reported motor success rates at 6 months, three at 12 months, one at 18 months, two at 24 months, and one at 36 months. Ten studies were conducted in China (697 patients) (Yu et al., 2024; Lang et al., 2019; Shi et al., 2021; Liu et al., 2025; Li et al., 2023; Zhang et al., 2024; Zhang et al., 2023; Qin et al., 2024; Wang et al., 2025; Qi et al., 2023), three in the United States (216 patients)(Nguyen et al., 2025; Cheung et al., 2024; Wan et al., 2017), and one in Thailand (114 patients) (Suwannaraj et al., 2023). Four studies reported the occurrence of permanent exotropia at 6 months, and seven studies evaluated stereopsis recovery. Among these, near stereopsis was assessed using the Titmus test in five studies (Lang et al., 2019; Zhang et al., 2024; Zhang et al., 2023; Qin et al., 2024; Wang et al., 2025), and two studies used random-dot stereograms (Yu et al., 2024; Liu et al., 2025). Distance stereopsis was evaluated using a synoptophore in four studies (Lang et al., 2019; Liu et al., 2025; Zhang et al., 2023; Wang et al., 2025). None of the included studies used electromyography (EMG) guidance for BTX injection. This distribution reflects the multicenter and cross-regional nature of the evidence base. In addition, all included studies achieved a NOS score of 7 or higher, indicating the overall high methodological quality of the evidence (Table 1).

### 3.2 Meta-analysis results

#### 3.2.1 Success rate results

**FIGURE 2:**
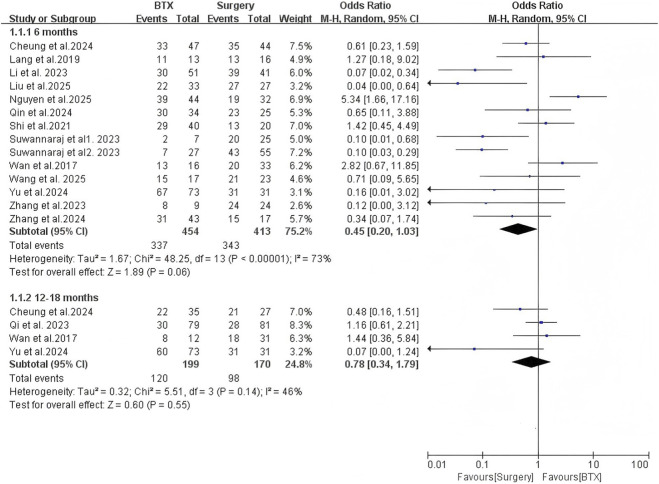
Forest plot comparing the motor success rate of BTX injection and surgery for AACE at different follow-up time.

Figure 2 compares the success rates of BTX injection and surgical treatment for AACE across different follow-up periods. The primary meta-analysis showed no statistically significant difference in success rates between the two treatments at 6 months (OR 0.45, 95% CI: 0.20–1.03, P = 0.06), with substantial heterogeneity (I^2^ = 73%, P_heterogeneity_<0.001). After excluding one study by Nguyen et al. (2025) through a leave-one-out sensitivity analysis, surgical treatment demonstrated a significantly higher success rate than BTX injection (OR 0.36, 95% CI: 0.17–0.77, P = 0.008), with moderate heterogeneity (I^2^ = 62%, P_heterogeneity_ = 0.002). In addition, no significant difference was observed between the two interventions at 12–18 months or 24–36months (12–18 months: OR 0.78, 95% CI: 0.34–1.79, P = 0.55, I^2^ = 46%, P_heterogeneity_ = 0.14; 24–36months: OR 0.29, 95%CI:0.03–3.31, P = 0.32, I^2^ = 86%, P_heterogeneity_<0.001). Notably, the analysis at 24–36 months demonstrated substantial heterogeneity and unstable results on sensitivity analysis. After exclusion of the study by Nguyen et al., the pooled estimate of the remaining two studies indicated a significant difference between interventions (OR 0.11, 95% CI: 0.02–0.52, P = 0.005, I^2^ = 17%, P_heterogeneity_ = 0.27) (Supplementary Figure S2).

#### 3.2.2 Stereopsis recovery rate results

**FIGURE 3:**
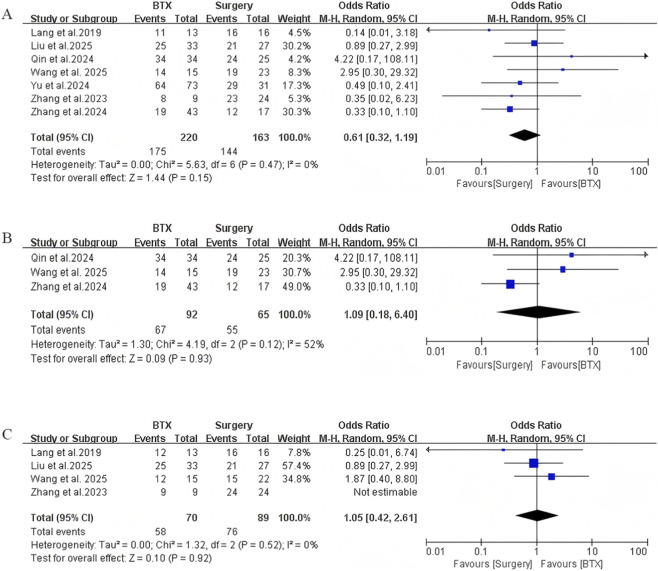
Forest plot of comparing the stereopsis recovery rate at 6 months following BTX injection and surgical treatment for AACE. **(A)** Overall results for near stereopsis (33 cm), **(B)** pediatric subgroup results for near stereopsis (33 cm), **(C)** overall results for distance stereopsis (6 m).

At 6 months, near stereopsis recovery was comparable between BTX and surgery in both the overall analysis and the children subgroup (overall: OR 0.61, 95% CI: 0.32–1.19, P = 0.15, I^2^ = 0%, P_heterogeneity_ = 0.47; children: OR 1.09, 95% CI: 0.18–6.40, P = 0.93, I^2^ = 52%, P_heterogeneity_ = 0.12). Distance stereopsis recovery was likewise similar between two groups (OR 1.05, 95% CI: 0.42–2.61, P = 0.92, I^2^ = 0%, P_heterogeneity_ = 0.52) (Figure 3).

#### 3.2.3 Incidence of permanent exotropia

**FIGURE 4:**
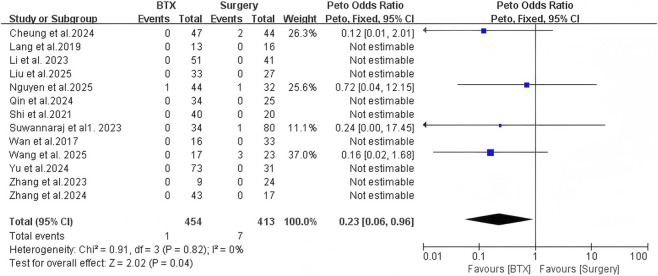
Forest plot of comparing the incidence of permanent exotropia at 6 months following BTX injection versus surgical treatment for AACE.

Nguyen et al. reported one case of consecutive exotropia in both the BTX and surgery groups, neither requiring further intervention. Additional cases of permanent exotropia were reported only in the surgical groups of the included studies, including 2 cases in Cheung et al., 1 case in Suwannaraj et al. and 3 cases in Wang et al. The pooled analysis using the Peto method demonstrated that the incidence of permanent exotropia was significantly lower in the BTX group than in the surgery group (Peto OR 0.23, 95% CI: 0.06–0.96, P = 0.04, I^2^ = 0%, P_heterogeneity_ = 0.82) (Figure 4).

#### 3.2.4 Subgroup analyses

##### 3.2.4.1 BTX injection method

**FIGURE 5:**
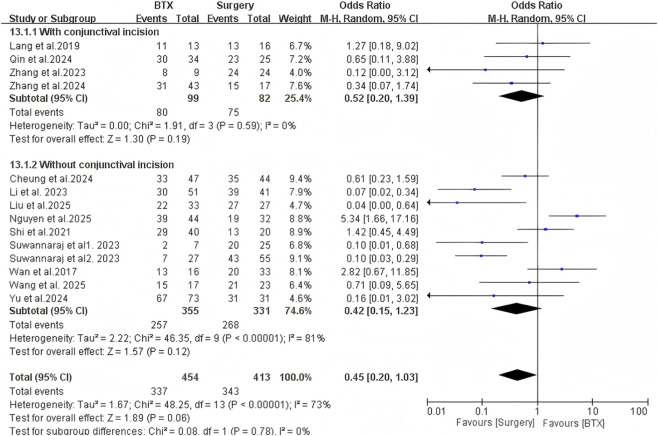
Forest plot comparing the 6-month motor success rate of BTX injection versus surgery for AACE based on whether BTX is injected via a conjunctival incision.

**FIGURE 6:**
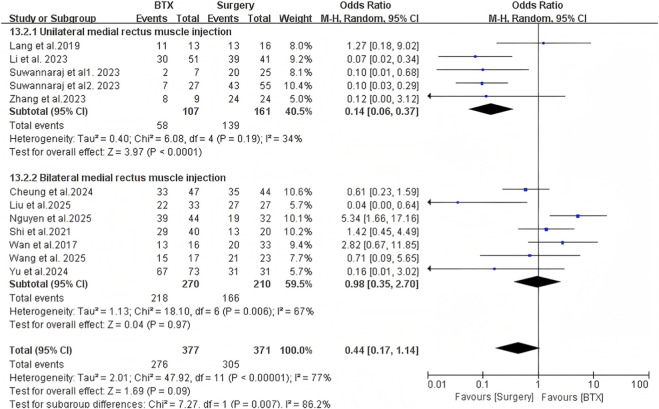
Forest plot comparing the 6-month motor success rate of BTX injection versus surgery for AACE based on unilateral or bilateral BTX injections.

Subgroup analysis based on whether BTX injection was performed with a conjunctival incision showed that, among studies using a conjunctival incision, there was no significant difference in efficacy between BTX injection and surgery (OR 0.52, 95% CI: 0.20–1.39, P = 0.19), with no heterogeneity (I^2^ = 0%, P = 0.59). Similarly, in studies without a conjunctival incision, BTX injection also showed no significant difference compared with surgery (OR 0.42, 95% CI: 0.20–1.03, P = 0.12), although substantial heterogeneity was found (I^2^ = 81%, P < 0.001) (Figure 5). Further subgroup analysis based on the injection method revealed different outcomes. Unilateral medial rectus injection was associated with lower efficacy than surgery (OR 0.14, 95% CI: 0.06–0.37, P < 0.001, I^2^ = 34%, P_heterogeneity_ = 0.19), whereas bilateral medial rectus injection achieved outcomes comparable to surgery (OR 0.98, 95% CI: 0.35–2.07, P = 0.97, I^2^ = 67%, P_heterogeneity_ = 0.006) (Figure 6).

##### 3.2.4.2 Pediatric subgroup

**FIGURE 7:**
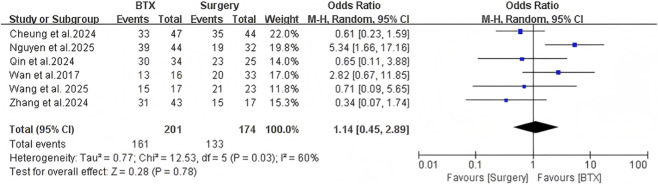
Forest plot of comparing the 6-month motor success rate of BTX injection versus surgery for AACE in pediatric patients.

Among pediatric patients with AACE, no significant difference in the 6-month motor success rate was observed between BTX injection and surgery (OR 1.14, 95% CI: 0.45–2.89, P = 0.78), with moderate heterogeneity (I^2^ = 60%, P = 0.03) (Figure 7).

##### 3.2.4.3 AACE duration

For AACE with a duration of less than 6 months, no statistically difference in success rates was found between BTX and surgery (OR 0.81, 95% CI: 0.33–1.98, P = 0.64, I^2^ = 60%, P_heterogeneity_ = 0.010). The initial pooled analysis of three studies with AACE durations exceeding 6 months revealed no significant difference in efficacy between BTX and surgery, with substantial heterogeneity (OR 0.28, 95% CI: 0.03–2.64, P = 0.27, I^2^ = 80%, P_heterogeneity_ = 0.006). Sensitivity analysis revealed that the study by Shi et al., 2021 substantially influenced the pooled estimate. After its exclusion, the remaining two studies demonstrated that BTX was significantly less effective than surgery (OR 0.09, 95% CI: 0.02–0.34, P < 0.001, I^2^ = 0%, P_heterogeneity_ = 0.63) (Supplementary Figure S3).

##### 3.2.4.4 Preoperative mean deviation

No significant difference was observed between BTX and surgery for moderate deviation (20–40 PD) (OR 0.64, 95% CI: 0.24–1.73, P = 0.38, I^2^ = 69%, P_heterogeneity_ = 0.001). The original pooled results from the three studies with a deviation greater than 40 PD did not show statistical significance between BTX and surgery (OR = 0.34, 95% CI: 0.10–1.15, P = 0.08, I^2^ = 55%, P_heterogeneity_ = 0.08). Sensitivity analysis indicated that the study by Lang et al., 2019 had a notable impact on the overall estimate. After sequentially removing this study, the combined findings of the remaining two studies showed that BTX was markedly inferior to surgery in efficacy (OR 0.23, 95% CI: 0.07–0.74, P = 0.01, I^2^ = 44%, P_heterogeneity_ = 0.17) (Supplementary Figure S4).

### 3.3 Assessment of sensitivity analysis and potential publication biases

**FIGURE 8:**
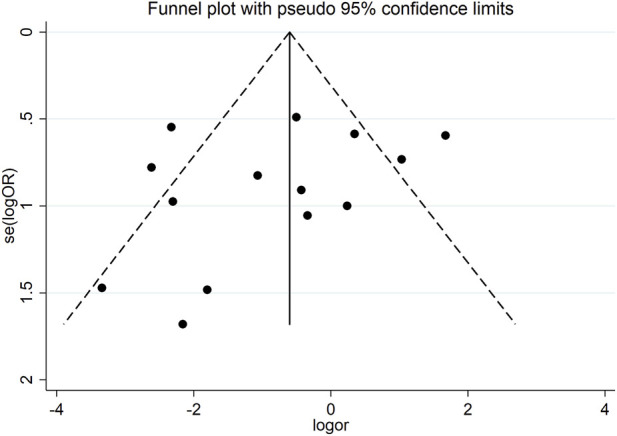
Funnel plot comparing BTX injection and surgical treatment for AACE at the 6-month follow-up.

Leave-one-out sensitivity analyses were performed to assess the robustness of the pooled estimates. Exclusion of the study by Nguyen et al. resulted in a statistically significant advantage for surgery (Supplementary Figure S1). Instability was also observed in the 24–36-month analysis and in subgroups with AACE duration greater than 6 months or large-angle deviation. Given that each subgroup included only three or four studies, these findings should be considered exploratory and interpreted cautiously. In addition, other sensitivity analyses in this study produced consistent and reliable results, further supporting the robustness of the remaining findings in this meta-analysis. Meta-regression revealed that age was a significant source of heterogeneity (P = 0.036) (Table 2). Finally, assessment of publication bias using Begg’s and Egger’s tests revealed no evidence of significant bias (Begg’s test: P = 0.443; Egger’s test: P = 0.322), enhancing confidence in the robustness and validity of the findings. The funnel plot was presented in Figure 8. Overall, despite residual unexplained heterogeneity, the synthesized evidence demonstrates acceptable methodological quality; however, the findings should be interpreted with caution given the limited number of studies in certain subgroup analyses.

**TABLE 2:** Meta-regression results.

Variables	Meta-regression	
	Coefficient (95%CI)	P value
BTX injection method	0.27 (-0.46, 0.99)	0.441
Preoperative-mean deviation	0.01 (-0.08, 0.09)	0.871
AACE duration	−0.65 (-3.17, 1.88)	0.587
Mean age	−0.11 (-0.20, −0.01)	**0.036**
Region	−0.15 (-1.40, 1.09)	0.791

Bold indicates the P value is less than 0.05.

## 4 Discussion

Multiple studies have compared the clinical outcomes of BTX injection with surgery for AACE, but their results remain contentious. Our meta-analysis included 14 studies and focused specifically on BTX single injection outcomes, providing more comprehensive evidence. At 6 months, pooled analysis showed no statistically significant difference between BTX and surgery in motor success rates. However, sensitivity analysis indicated that the study by Nguyen et al. (2025) had a substantial influence on the motor results at 6 months, as exclusion of this study shifted the result in favor of surgery. This may be attributed to the markedly lower surgical success rate reported in that study, likely driven by the inclusion of 73.7% of participants younger than 5 years. Nevertheless, Nguyen et al. (2025) was retained in the final meta-analysis because of its strong methodological quality, non-inferiority design, and status as the largest available study focusing on pediatric AACE. Subgroup analyses suggested that outcomes were more stable and comparable in pediatric patients, in bilateral medial rectus injections, and in patients with moderate deviation or shorter disease duration. Therefore, while the estimate of 6-month motor success should be interpreted with caution, the available evidence still suggests potentially comparable short-term outcomes between BTX and surgery in selected patients, particularly in pediatric patients. Additionally, sensory outcomes were also similar between the two treatments, and the incidence of permanent exotropia after BTX injection remained low. At 12–18 months, limited available evidence suggested similar motor outcomes between BTX and surgery.

Substantial heterogeneity was observed in the 6-month motor outcomes, which is not unexpected in a meta-analysis predominantly based on observational studies. Meta-regression identified age as a significant contributor, explaining approximately 31.54% of the between-study heterogeneity. However, a substantial proportion remained unexplained, suggesting the influence of additional clinical and methodological factors. First, BTX dosing strategies varied markedly across studies and may have substantially influenced treatment response. The included studies used different dosing strategies, with some adopting fixed-dose regimens and others individualizing BTX dosage according to the preoperative angle of deviation. Specifically, for patients with deviations <20 PD, administered doses ranged from 1.25 to 4 IU; for deviations between 20 and 40 PD, doses ranged from 2 to 5 IU; and for deviations between 40 and 60 PD, doses ranged from 4 to 7.5 IU. Notably, among patients with deviations >60 PD, one study used a dose of 2.5 IU whereas another used 7 IU. These findings indicate that patients with comparable disease severity may have received markedly different levels of chemodenervation across studies. Importantly, Zhang et al. reported significantly lower success rates with 2.5 IU compared with 5 IU in large-angle AACE, supporting a dose-dependent treatment effect of BTX. Therefore, variation in dosing strategies may have influenced both treatment efficacy and the risk of overcorrection, contributing to between-study heterogeneity. Nevertheless, due to inconsistent reporting and the limited number of studies within individual dosing categories, dose-standardized subgroup analyses were not feasible, representing an important limitation of this study. Second, variation in motor success definitions may also have contributed to heterogeneity and influenced comparisons between BTX and surgery. Although most included studies defined motor success as postoperative deviation <10 PD, two studies used a stricter threshold of <5 PD (Wang et al., 2025; Yu et al., 2024), and one study used <8 PD (Li et al., 2023). Because surgical correction permits intraoperative adjustment, whereas BTX cannot be adjusted during the procedure, stricter motor success definitions are likely to disproportionately disadvantage BTX in comparative analyses. Consistent with this possibility, studies using stricter success criteria generally reported lower relative motor success rates for BTX compared with the overall pooled estimate. Nevertheless, sensitivity analyses showed that inclusion of these studies did not materially alter the direction of the pooled effect estimates, supporting the rationale for their inclusion despite the use of stricter outcome thresholds.

Third, baseline imbalances between treatment groups likely represented another major contributor to heterogeneity. Yu et al. reported a markedly longer disease duration in the surgery group, while Shi et al. reported significantly larger preoperative deviation angles among surgically treated patients. Zhang et al. and Qin et al. also stratified treatment selection according to disease duration. These differences partly stem from the inherent selection bias of retrospective studies, and are also closely linked to the clinical features and treatment patterns characteristic of AACE. In practice, ocular deviation in AACE often stabilizes after approximately 6 months, which is commonly considered the appropriate timing for surgery. In contrast, BTX injection can be administered during earlier disease stages to relieve diplopia and restore binocular alignment. Moreover, in patients with longer disease duration or larger deviation angles, clinicians may preferentially select surgery because of concerns regarding recurrence risk and potential dose-related complications associated with BTX. Consequently, this treatment selection pattern based on disease severity and clinical judgment may have introduced directional confounding, potentially underestimating the efficacy of surgery or overestimating the effectiveness of BTX. To further explore the influence of baseline imbalance, we performed subgroup analyses stratified by preoperative deviation angle and disease duration. BTX achieved outcomes similar to surgery in patients with moderate-angle deviation or shorter disease duration. In contrast, substantial heterogeneity and unstable sensitivity analyses were observed in subgroups with disease duration exceeding 6 months and large-angle deviation, suggesting that more advanced disease severity may have contributed importantly to between-study variability and inconsistent treatment effects. Additionally, geographic variation may have further influenced heterogeneity. Of the included studies, ten were conducted in China, three in the United States, and one in Thailand. Differences in referral patterns, surgical techniques, BTX injection methods, and treatment preferences across regions may have influenced both patient selection and treatment outcomes. Although meta-regression did not identify geographic region as a statistically significant source of heterogeneity, the predominance of studies from a single country may still limit the generalizability of the findings. Taken together, these findings suggest that heterogeneity in the present study is multifactorial and reflects both clinical and methodological variability across studies. Although several potential contributors were identified and explored, residual heterogeneity remains unavoidable and should be carefully considered when interpreting the pooled estimates.

The therapeutic effects of BTX in AACE may be mediated by several complementary mechanisms. BTX rapidly reduces medial rectus overactivity by inhibiting acetylcholine release at the neuromuscular junction (Xu et al., 2020), restoring the force balance between the agonist and antagonist extraocular muscles and facilitating early ocular realignment. Although its pharmacologic effect is temporary, the improvement in ocular alignment may persist beyond the duration of chemodenervation (Issaho et al., 2017; Erickson et al., 2015; Croes et al., 2007). One possible explanation is that transient weakening of the medial rectus muscle facilitates the restoration of binocular fusion and sensory adaptation, which may persist even after the pharmacologic effect subsides (Niyaz et al., 2023; Gursoy et al., 2012; Scott, 1980). This mechanism may be particularly relevant in AACE, which typically develops abruptly in patients with previously normal binocular vision. The preservation of premorbid binocular function may provide a favorable neural basis for the recovery and maintenance of binocular alignment following BTX treatment (Yu et al., 2024). These neurophysiological effects may also help explain age-dependent differences in treatment response. In pediatric patients, both motor and sensory outcomes following BTX injection appear comparable to those of surgery, potentially reflecting greater neural plasticity that facilitates binocular reorganization and muscular adaptation after transient pharmacologic paralysis (Pawar et al., 2025). Early intervention in children may further reduce binocular suppression and promote recovery of stereopsis. From a practical perspective, BTX also offers advantages in this population, including shorter anesthesia exposure, reduced procedural time, and potentially lower cost. In addition, BTX preserves extraocular muscle anatomy and may reduce the risk of surgically induced permanent exotropia (Koçkar et al., 2025; Reche-Sainz et al., 2025). Taken together, these features support BTX as a reasonable alternative to surgery in appropriately selected pediatric patients. However, several studies have reported declining BTX efficacy with increasing age, particularly in older teenagers or adults, where reduced neural adaptability may limit sustained binocular recovery (Shi et al., 2021; Yu et al., 2025). Zhang et al. further identified older age as a significant risk factor for recurrence after BTX treatment (Zhang et al., 2025). Because direct comparative evidence in adult populations remains limited, further studies are needed to clarify the influence of age on treatment response.

In additon, subgroup analyses suggest injection technique may influence comparisons between BTX and surgical outcomes, consistent with previous findings (McNeer and Tucker 2010; Yu et al., 2025; Kang et al., 2025). Unilateral medial rectus injection appeared less effective than surgery, whereas bilateral injection demonstrated outcomes more comparable to surgical treatment. Bilateral chemodenervation may produce a more balanced reduction in medial rectus activity in both eyes, facilitating the re-establishment of binocular alignment by minimizing interocular motor asymmetry (Scott 1980). In AACE, where disruption of fusional vergence mechanisms is a crucial pathophysiological feature, restoring symmetry in ocular motor input is particularly important for achieving stable binocular fusion (Li et al., 2026). In contrast, unilateral injection may produce asymmetric weakening of convergence tone, which may be insufficient to fully rebalance vergence control and limit the restoration of ocular alignment. These findings suggest that bilateral injection may confer a potential advantage when BTX is used to treat AACE. However, given the limited number of studies and the lack of direct comparative trials, further prospective studies are needed to clarify the role of injection laterality in AACE. Regarding conjunctival incision, our results showed no significant difference in success rates between injections performed with or without an incision. Direct visualization of the medial rectus may facilitate more precise placement of the toxin (Wan et al., 2011), but creating a conjunctival incision may introduce additional risks, such as iatrogenic scarring and longer anesthesia time. Overall, the use of conjunctival incision varied across studies, likely reflecting differences in surgical practice and surgeon preference.

In terms of complications, adverse effects of BTX injection mainly include ptosis, exotropia, and vertical strabismus, which are typically attributable to transient paralysis caused by diffusion of the toxin to adjacent muscles (Huang et al., 2022; Ricci et al., 2015). In the studies included in our analysis, BTX-related complications were temporary and typically resolved spontaneously within 1–3 months, without lasting effects on ocular alignment or visual function. In contrast, surgical complications include postoperative conjunctival hemorrhage, conjunctival incision scarring, and consecutive exotropia, the latter of which can significantly impact postoperative satisfaction and visual function. Owing to its pharmacological reversibility, BTX injection may reduce the risk of permanent exotropia compared with surgery. Among the included studies, only one study reported a case of consecutive exotropia after BTX injection that required no further management (Nguyen et al., 2025), whereas permanent exotropia was described in four surgical cohorts (Cheung et al., 2024; Suwannaraj et al., 2023; Wang et al., 2025; Nguyen et al., 2025). Similarly, our meta-analysis demonstrated that BTX may led to a lower incidence of permanent exotropia compared with surgery, suggesting its potential safety advantage. However, given the limited number of studies and small sample sizes, this finding is preliminary and hypothesis-generating, and should be confirmed in adequately powered prospective studies before definitive safety conclusions are made.

Only a limited number of studies have evaluated the mid- and long-term outcomes of BTX versus surgery for AACE. Current evidence suggests that the two treatment modalities may have comparable outcomes at mid-term follow-up; however, this finding is limited by the small number of retrospective studies and should be interpreted with low certainty. Regarding long-term outcomes, only three studies are currently available, and these exhibit a degree of heterogeneity. Specifically, the study by Cheng et al. reported a loss to follow-up exceeding 50% beyond 24 months, potentially undermining the reliability of long-term outcomes. In addition, the study by Yu et al. included both pediatric and adult patients, whereas the study by Nguyen et al. focused exclusively on pediatric populations, resulting in substantial heterogeneity across study cohorts. Consequently, definitive conclusions regarding the long-term efficacy of BTX compared with surgery cannot be drawn. Previous studies have suggested that BTX injection may induce not only temporary chemodenervation but also secondary sensory and motor adaptations, which could potentially influence ocular alignment over time (Gursoy et al., 2012; Rattanalert et al., 2025). To better understand these effects and clarify the long-term efficacy of BTX in AACE, additional high-quality studies with extended follow-up are warranted.

There are several limitations to this meta-analysis. First, the number of eligible studies was limited and substantial clinical heterogeneity was observed in overall 6-month motor outcomes. Second, most included studies were observational, introducing the risk of bias inherent to non-randomized designs. Third, none of the included studies used EMG-guided injection, which may reduce injection precision and therefore limit the generalizability of the findings. Finally, our analysis was restricted to a single BTX injection to improve comparability across studies and reduce methodological heterogeneity. However, this approach may underestimate the overall effectiveness of BTX in real-world clinical practice, where repeated injections are often used to achieve and maintain optimal ocular alignment. Future high-quality studies with long-term follow-up are needed to directly compare repeated BTX injections with surgical treatment to better reflect real-world clinical outcomes.

## 5 Conclusion

Overall, the pooled short-term motor outcome showed substantial heterogeneity and sensitivity to individual studies. More consistent findings were observed in pediatric populations and in studies using bilateral medial rectus injections, where BTX may achieve short-term outcomes comparable to surgery. Stereopsis recovery at 6 months was also similar between the two treatments. However, given the limited number of available studies and the predominance of retrospective designs, adequately powered RCTs and prospective studies are needed to establish more definitive conclusions.

## Data Availability

The original contributions presented in the study are included in the article/Supplementary Material, further inquiries can be directed to the corresponding authors.
