# Supplementary material for: Comparative efficacy of botulinum toxin and surgical treatment for acute acquired concomitant esotropia: a systematic review and meta-analysis
Source: Front Pharmacol. 2026 Jun 17;17:1775497. doi: 10.3389/fphar.2026.1775497 (PMC13318883; doi:10.3389/fphar.2026.1775497)
Supplement: Supplementary file 1 [file Supplementaryfile1.docx]

**The supplementary description**

Supplemental Table S1. PRISMA _2020_Checklist.

Supplemental Table S2. PubMed literature search strategy, adapted for EMBASE, Web of Science, the Cochrane Library and China Biomedical Sinomed

Supplemental Table S3. Summary of the baseline characteristics and success rates across all 14 studies.

Supplemental Figure S1. Sensitivity analysis comparing BTX injection and surgical treatment for AACE at the 6-month follow-up.

Supplemental Figure S2. Forest plot comparing the motor success rate of BTX injection and surgery for AACE at 24-36 months.

Supplemental Figure S3. Forest plot of subgroup analysis based on AACE duration.

Supplemental Figure S4. Forest plot of subgroup analysis based on AACE preoperative mean deviation.

Supplemental Table S1 PRISMA _2020_Checklist.

| **S1 PRISMA _2020_Checklist** | | | |
| --- | --- | --- | --- |
| **Section and Topic** | **Item #** | **Checklist item** | **Location where item is reported** |
| **TITLE** | | |  |
| Title | 1 | Identify the report as a systematic review. | Title |
| **ABSTRACT** | | |  |
| Abstract | 2 | See the PRISMA 2020 for Abstracts checklist. | Abstract |
| **INTRODUCTION** | | |  |
| Rationale | 3 | Describe the rationale for the review in the context of existing knowledge. | 1 Introduction |
| Objectives | 4 | Provide an explicit statement of the objective(s) or question(s) the review addresses. |  |
| **METHODS** | | |  |
| Eligibility criteria | 5 | Specify the inclusion and exclusion criteria for the review and how studies were grouped for the syntheses. | 2.2 Inclusion and Exclusion Criteria |
| Information sources | 6 | Specify all databases, registers, websites, organisations, reference lists and other sources searched or consulted to identify studies. Specify the date when each source was last searched or consulted. | 2.1 Search Strategy |
| Search strategy | 7 | Present the full search strategies for all databases, registers and websites, including any filters and limits used. |  |
| Selection process | 8 | Specify the methods used to decide whether a study met the inclusion criteria of the review, including how many reviewers screened each record and each report retrieved, whether they worked independently, and if applicable, details of automation tools used in the process. | 2.3 Data Extraction and Quality Evaluation |
| Data collection process | 9 | Specify the methods used to collect data from reports, including how many reviewers collected data from each report, whether they worked independently, any processes for obtaining or confirming data from study investigators, and if applicable, details of automation tools used in the process. |  |
| Data items | 10a | List and define all outcomes for which data were sought. Specify whether all results that were compatible with each outcome domain in each study were sought (e.g. for all measures, time points, analyses), and if not, the methods used to decide which results to collect. |  |
|  | 10b | List and define all other variables for which data were sought (e.g. participant and intervention characteristics, funding sources). Describe any assumptions made about any missing or unclear information. |  |
| Study risk of bias assessment | 11 | Specify the methods used to assess risk of bias in the included studies, including details of the tool(s) used, how many reviewers assessed each study and whether they worked independently, and if applicable, details of automation tools used in the process. | 2.4 Statistical Analysis |
| Effect measures | 12 | Specify for each outcome the effect measure(s) (e.g. risk ratio, mean difference) used in the synthesis or presentation of results. |  |
| Synthesis methods | 13a | Describe the processes used to decide which studies were eligible for each synthesis (e.g. tabulating the study intervention characteristics and comparing against the planned groups for each synthesis (item #5)). |  |
|  | 13b | Describe any methods required to prepare the data for presentation or synthesis, such as handling of missing summary statistics, or data conversions. |  |
|  | 13c | Describe any methods used to tabulate or visually display results of individual studies and syntheses. |  |
|  | 13d | Describe any methods used to synthesize results and provide a rationale for the choice(s). If meta-analysis was performed, describe the model(s), method(s) to identify the presence and extent of statistical heterogeneity, and software package(s) used. |  |
|  | 13e | Describe any methods used to explore possible causes of heterogeneity among study results (e.g. subgroup analysis, meta-regression). |  |
|  | 13f | Describe any sensitivity analyses conducted to assess robustness of the synthesized results. |  |
| Reporting bias assessment | 14 | Describe any methods used to assess risk of bias due to missing results in a synthesis (arising from reporting biases). |  |
| Certainty assessment | 15 | Describe any methods used to assess certainty (or confidence) in the body of evidence for an outcome. |  |
| **RESULTS** | | |  |
| Study selection | 16a | Describe the results of the search and selection process, from the number of records identified in the search to the number of studies included in the review, ideally using a flow diagram. | 3.1 Characteristics of the studies |
|  | 16b | Cite studies that might appear to meet the inclusion criteria, but which were excluded, and explain why they were excluded. |  |
| Study characteristics | 17 | Cite each included study and present its characteristics. |  |
| Risk of bias in studies | 18 | Present assessments of risk of bias for each included study. | 3.3 Assessment of sensitivity analysis and potential publication biases |
| Results of individual studies | 19 | For all outcomes, present, for each study: (a) summary statistics for each group (where appropriate) and (b) an effect estimate and its precision (e.g. confidence/credible interval), ideally using structured tables or plots. | 3.2 Meta-analysis results |
| Results of syntheses | 20a | For each synthesis, briefly summarise the characteristics and risk of bias among contributing studies. |  |
|  | 20b | Present results of all statistical syntheses conducted. If meta-analysis was done, present for each the summary estimate and its precision (e.g. confidence/credible interval) and measures of statistical heterogeneity. If comparing groups, describe the direction of the effect. |  |
|  | 20c | Present results of all investigations of possible causes of heterogeneity among study results. |  |
|  | 20d | Present results of all sensitivity analyses conducted to assess the robustness of the synthesized results. |  |
| Reporting biases | 21 | Present assessments of risk of bias due to missing results (arising from reporting biases) for each synthesis assessed. | 3.3 Assessment of sensitivity analysis and potential publication biases |
| Certainty of evidence | 22 | Present assessments of certainty (or confidence) in the body of evidence for each outcome assessed. | 3.2 Meta-analysis results  3.3 Assessment of sensitivity analysis and potential publication biases |
| **DISCUSSION** | | |  |
| Discussion | 23a | Provide a general interpretation of the results in the context of other evidence. | 4 Discussion |
|  | 23b | Discuss any limitations of the evidence included in the review. |  |
|  | 23c | Discuss any limitations of the review processes used. |  |
|  | 23d | Discuss implications of the results for practice, policy, and future research. |  |
| **OTHER INFORMATION** | | |  |
| Registration and protocol | 24a | Provide registration information for the review, including register name and registration number, or state that the review was not registered. | 2.1 Search Strategy |
|  | 24b | Indicate where the review protocol can be accessed, or state that a protocol was not prepared. |  |
|  | 24c | Describe and explain any amendments to information provided at registration or in the protocol. | Not applicable |
| Support | 25 | Describe sources of financial or non-financial support for the review, and the role of the funders or sponsors in the review. | Funding |
| Competing interests | 26 | Declare any competing interests of review authors. | Conflict of Interest |
| Availability of data, code and other materials | 27 | Report which of the following are publicly available and where they can be found: template data collection forms; data extracted from included studies; data used for all analyses; analytic code; any other materials used in the review. | Data Availability Statement |

Supplemental Table S2. PubMed literature search strategy, adapted for EMBASE, Web of Science, the Cochrane Library and China Biomedical Sinomed

| **Number** | **Search Terms** |
| --- | --- |
| 1 | esotropia[MeSH Terms] |
| 2 | acute acquired concomitant esotropia[Title/Abstract] OR acute concomitant esotropia[Title/Abstract] OR concomitant esotropia[Title/Abstract] |
| 3 | Botulinum Toxins[MeSH Terms] |
| 4 | Toxins, Botulinum[Title/Abstract] OR Botulinum Neurotoxin[Title/Abstract] OR Neurotoxin, Botulinum[Title/Abstract] OR Botulin[Title/Abstract] OR Botulinum Toxin[Title/Abstract] OR Toxin, Botulinum[Title/Abstract] OR Clostridium botulinum Toxins[Title/Abstract] OR Toxins, Clostridium botulinum[Title/Abstract] OR Botulinum Neurotoxins[Title/Abstract] OR Neurotoxins, Botulinum[Title/Abstract] |
| 5 | surgery[MeSH Terms] |
| 6 | (esotropia[MeSH Terms]) OR (acute acquired concomitant esotropia[Title/Abstract] OR acute concomitant esotropia[Title/Abstract] OR concomitant esotropia[Title/Abstract]) |
| 7 | (Botulinum Toxins[MeSH Terms]) OR (Toxins, Botulinum[Title/Abstract] OR Botulinum Neurotoxin[Title/Abstract] OR Neurotoxin, Botulinum[Title/Abstract] OR Botulin[Title/Abstract] OR Botulinum Toxin[Title/Abstract] OR Toxin, Botulinum[Title/Abstract] OR Clostridium botulinum Toxins[Title/Abstract] OR Toxins, Clostridium botulinum[Title/Abstract] OR Botulinum Neurotoxins[Title/Abstract] OR Neurotoxins, Botulinum[Title/Abstract]) |
| 8 | (((strabismus surgery[Title/Abstract]) OR (Extraocular muscle surgery[Title/Abstract])) OR (medial rectus recession[Title/Abstract])) OR (lateral rectus recession[Title/Abstract])) OR (recession-resection[Title/Abstract])) OR (muscle transposition[Title/Abstract])) OR (resection[Title/Abstract])) OR (recession[Title/Abstract]))) |
| 9 | (surgery[MeSH Terms]) OR (((strabismus surgery[Title/Abstract]) OR (Extraocular muscle surgery[Title/Abstract])) OR (medial rectus recession[Title/Abstract])) OR (lateral rectus recession[Title/Abstract])) OR (recession-resection[Title/Abstract])) OR (muscle transposition[Title/Abstract])) OR (resection[Title/Abstract])) OR (recession[Title/Abstract]))) |
| 10 | ((esotropia[MeSH Terms]) OR (acute acquired concomitant esotropia[Title/Abstract] OR acute concomitant esotropia[Title/Abstract] OR concomitant esotropia[Title/Abstract] OR AACE[Title/Abstract])) AND (((Botulinum Toxins[MeSH Terms]) OR (Toxins, Botulinum[Title/Abstract] OR Botulinum Neurotoxin[Title/Abstract] OR Neurotoxin, Botulinum[Title/Abstract] OR Botulin[Title/Abstract] OR Botulinum Toxin[Title/Abstract] OR Toxin, Botulinum[Title/Abstract] OR Clostridium botulinum Toxins[Title/Abstract] OR Toxins, Clostridium botulinum[Title/Abstract] OR Botulinum Neurotoxins[Title/Abstract] OR Neurotoxins, Botulinum[Title/Abstract])) OR ((((((((strabismus surgery[Title/Abstract]) OR (extraocular muscle surgery[Title/Abstract])) OR (medial rectus recession[Title/Abstract])) OR (lateral rectus recession[Title/Abstract])) OR (recession-resection[Title/Abstract])) OR (muscle transposition[Title/Abstract])) OR (resection[Title/Abstract])) OR (recession[Title/Abstract]))) |

Table S3 Summary of the baseline characteristics and success rates across all 14 studies.

| Study | Country | Age, Mean/Range, y | Duration of AACE | Angle range (PD) | | | Sample size, N,  BTX/Surgery | Definition of motor success | BTX injection dose | BTX injection method | Motor success rate at 6 months(%, follow-up time), BTX/Surgery | Stereopsis recovery rate at 6 months (%), BTX/Surgery |
| --- | --- | --- | --- | --- | --- | --- | --- | --- | --- | --- | --- | --- |
|  |  |  |  | Near | Distance | |  |  |  |  |  |  |
| Yu et al. 2024 | China | BTX: 29.8  Surgery: 29.35 | BTX: 1.53±2.01 y  Surgery: 4.34±2.84 y*****  **Surgery > BTX** | BTX: 24.51±13.31  Surgery: 28.39±13.75 | BTX: 25.86±13.38  Surgery: 35.16±13.69*  **Surgery > BTX** | | 73/31 | **≤5PD** | 3.5IU:16–20PD  4IU:21–40PD 4.5IU:40–50PD | M2 | 91.98/100 (6 months)  82.19/100(12 months)  **68.49/100(24 months)*** | 87.67/93.55 (near) |
| Nguyen et al. 2025 | USA | Overall: 2-10 | BTX: 2.5(0.5-18.0) m  Surgery: 7.0(2.0-13.0) m*  **Surgery > BTX** | NR | BTX: 35(10-55)  Surgery: 35(12-55) | | 44/32 | ≤10PD | 5(3.75-5) IU | M2 | **88.64/59.38 (6 months)***  72.73/56.25(36 months) | NR |
| Liu et al. 2025 | China | BTX: 8-54  Surgery: 6-56 | Overall: ≤6 m | BTX: 30(20-40)  Surgery: 30(20-40) | BTX: 30(20-40)  Surgery: 30(20-40) | | 33/27 | ≤10PD | 2.5IU:<30PD  5IU:>30PD | M2 | **66.67/100 (6 months)*** | 75.56/77.78(near & distance) |
| Cheung et al. 2024 | USA | Overall: 2-17 | BTX: 4.43(2.2,6.1) m  Surgery: 4.93(2.87,7.1) m | BTX: 35(30, 45)  Surgery: 35(30, 45) | BTX: 35(25, 40)  Surgery: 35(30, 40) | | 47/44 | ≤10PD | 5(2.5-7.5) IU | M2 | 70.21/79.55 (6 months)  62.86/77.78 (12 months)  52/86.36 (24 months) | NR |
| Suwannaraj et al 1. 2023 | Thailand | Overall: 5-59 | NR | NR | BTX: 30.7±11  Surgery: 30.0±8.5 | | 7/25 | ＜10PD | 5IU | M1 | **28.57/80 (6 months)*** | NR |
| Suwannaraj et al 2. 2023 | Thailand | Overall: 5-59 | NR | NR | BTX: 56.5±9.4  Surgery: 60.3±12.7 | | 27/55 | ＜10PD | 5IU | M1 | **25.93/78.18 (6 months)*** | NR |
| Li et al. 2023 | China | Overall: 6-50 | Overall: 1(0.08-10) y | BTX: 18(12.5-25)  Surgery: 35(25-40)*  **Surgery > BTX** | BTX: 20 (15-30)  Surgery: 40 (25-45)*  **Surgery > BTX** | | 51/41 | **＜8PD** | 3IU:10–25PD  3.5IU:26–35PD  4IU:>35PD | M1 | **58.82/95.12 (6 months)*** | NR |
| Shi et al. 2021 | China | BTX: 22.4  Surgery: 18.0 | BTX: 9.60±10.2 m  Surgery: 16.5±13.5 m | BTX:27.1±13.8  Surgery: 39.6±11.0 | BTX: 32.3±15.4  Surgery: 44.0±11.4 | | 40/20 | ＜10PD | 4IU: <30PD  5IU:30-40PD  6IU:40-50PD  7IU:>60PD | M2 | 72.5/65 (6 months) | NR |
| Lang et al. 2019 | China | BTX: 3-24  Surgery: 3-32 | BTX: 3.38±1.7 m  Surgery: 2.31±1.35 m | BTX: 41.92±27.65  Surgery: 32.66±24.11 | BTX: 47.30±22.69  Surgery: 39.66±20.13 | | 13/16 | ≤10PD | 2.5IU | M3 | 84.62/81.25 (6 months) | 84.62/100(near)  92.31/100(distance) |
| Wan et al. 2017 | USA | Overall: 2-10 | BTX: 3.0(0.5-12.7) m  Surgery: 6.8 (2.1-12.7) m | NR | BTX: 35(10-55)  Surgery: 35(12-55) | | 16/33 | ≤10PD | 5IU | M2 | 84.62/81.25 (6 months)  66.67/58.06 (18months) | NR |
| Zhang et al. 2024 | China | 2.5IU BTX: 4-6  5.0IU BTX: 3-8.5  Surgery: 4-9 | 2.5IU BTX: 0.29(0.18-0.47)y  5IU BTX: 0.33(0.20-0.50)y  Surgery: 0.75(0.5-2.0) y*  **Surgery > BTX** | 2.5 IU BTX: 61.25(59.38-70.63)  5.0 IU BTX: 67.50(60.00-70.00)  Surgery: 61.25(55.63-70.00) | | | 43/17 | ＜10PD | 2.5/5 IU | M5 | 72.09/88.24 (6 months) | 44.19/70.59(near) |
| Zhang et al. 2023 | China | Overall: 4-49 | BTX: ＜6m  Surgery: ≥6m | BTX: 36.89±13.79  Surgery: 37.94±19.58 | | | 9/24 | ≤10PD | 2IU:<20PD  3IU:20–35PD  4IU:35–50PD  5IU:>50PD | M3 | 88.89/100 (6 months) | 88.89/95.83(near)  100/100(distance) |
| Qin et al. 2024 | China | Overall: 6-14 | BTX: ＜6m  Surgery: ≥6m  **Surgery > BTX** | BTX: 36.72±12.67  Surgery: 37.24±13.85 | | | 34/25 | ＜10PD | 4-5 IU | M5 | 88.24/92 (6 months) | 100/96(near) |
| Wang et al. 2025 | China | Overall: 3-18 | BTX: 4.18±5.05 m  Surgery: 9.57±7.38 m*  **Surgery > BTX** | BTX: 41.35±16.90  Surgery:49.2±18.25 | | BTX: 39.71±14.94  Surgery: 47.0±18.53 | 17/23 | **＜5PD** | 2.5IU:<50PD  5IU:>50PD | M2 | 88.24/91.3 (6 months) | 93.33/82.61(near)  80/68.18(distance) |
| Qi et al. 2023 | China | BTX: 31.53  Surgery: 32.64 | BTX: 12.64±3.46 y  Surgery: 12.38±3.76 y | BTX: 26.91±5.74  Surgery: 27.35±5.79 | | | 79/81 | ＜10PD | 1.25-2.5IU:<20PD  2.5-3.5IU:20-40PD  2.5-5IU:>40PD | M3 | 37.97/34.57(12months) | NR |

*: The BTX group showed a statistically significant difference compared to the surgery group, P＜0.05. M1: Uilateral medial rectus muscle injection without conjunctival incision. M2: Bilateral medial rectus muscle injection without conjunctival incision. M3: Unilateral medial rectus muscles with a conjunctival incision. M4: Bilateral medial rectus muscles with a conjunctival incision. M5: Unilateral or bilateral medial rectus muscles with a conjunctival incision. NR: not reported.

Figure S1. Sensitivity analysis of the overall 6-month motor success rate comparing BTX injection and surgery for AACE.


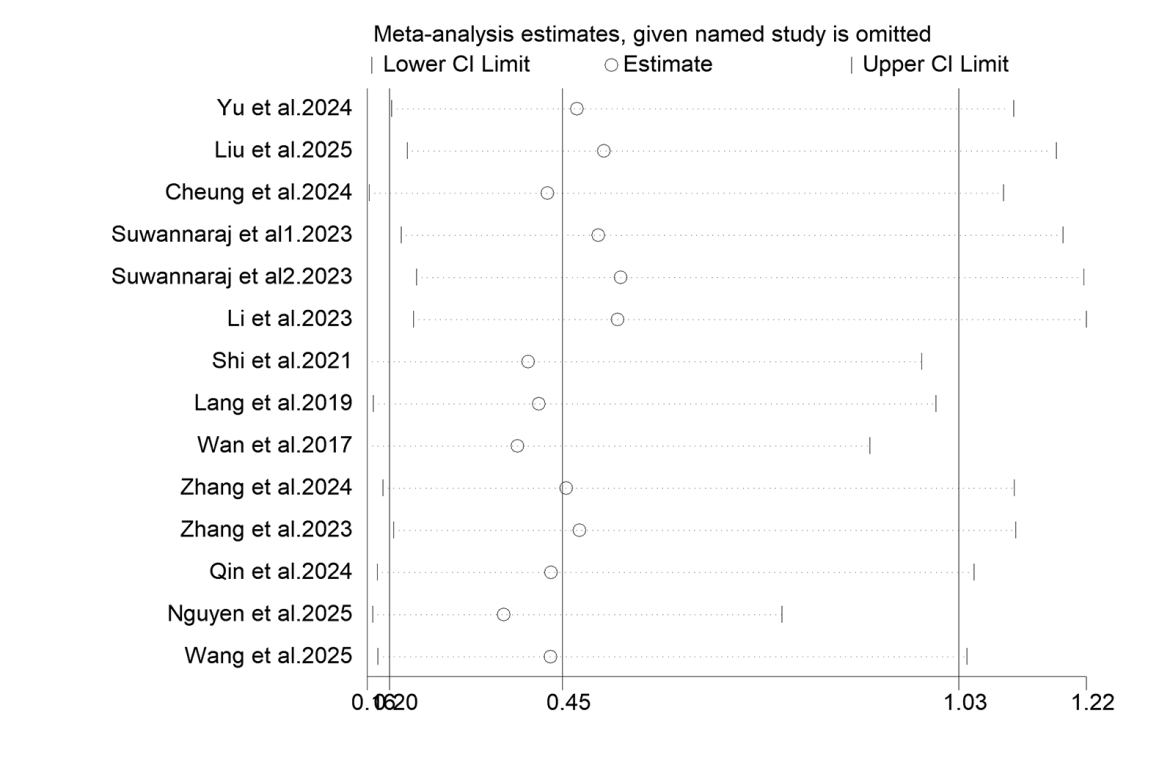


1. Results including Nguyen et al.


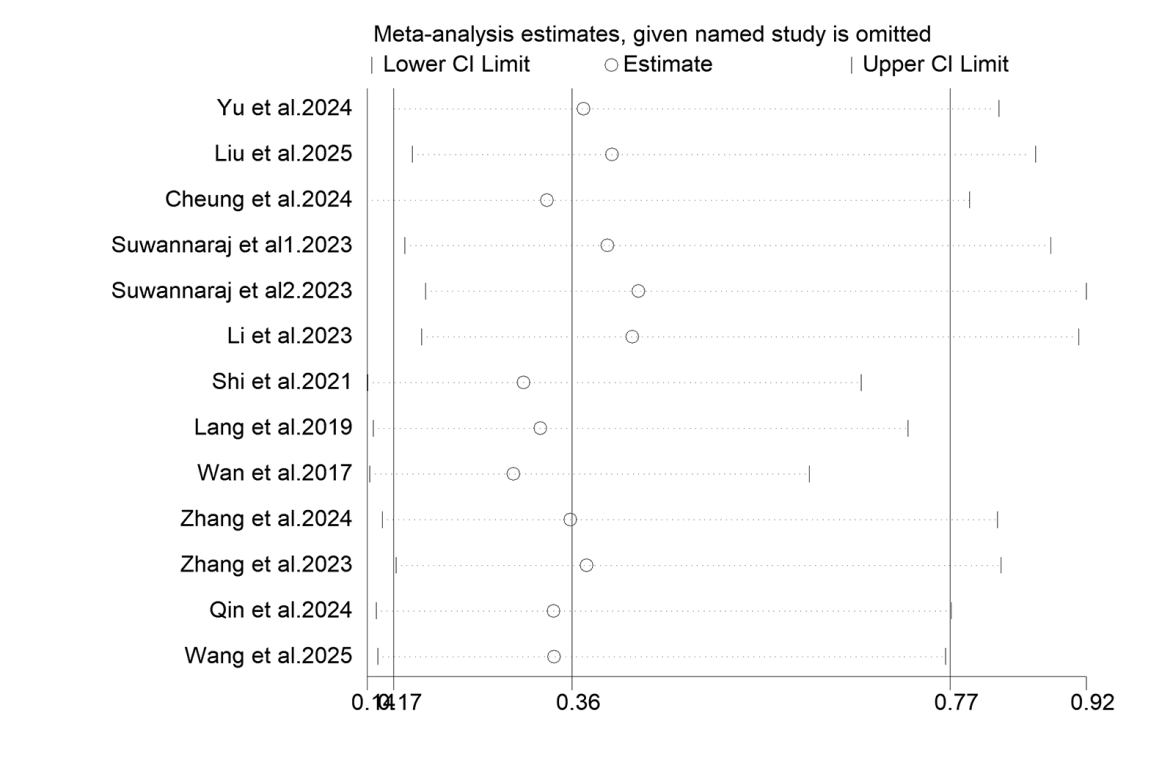


1. Results excluding Nguyen et al. to assess its impact on the pooled estimate.


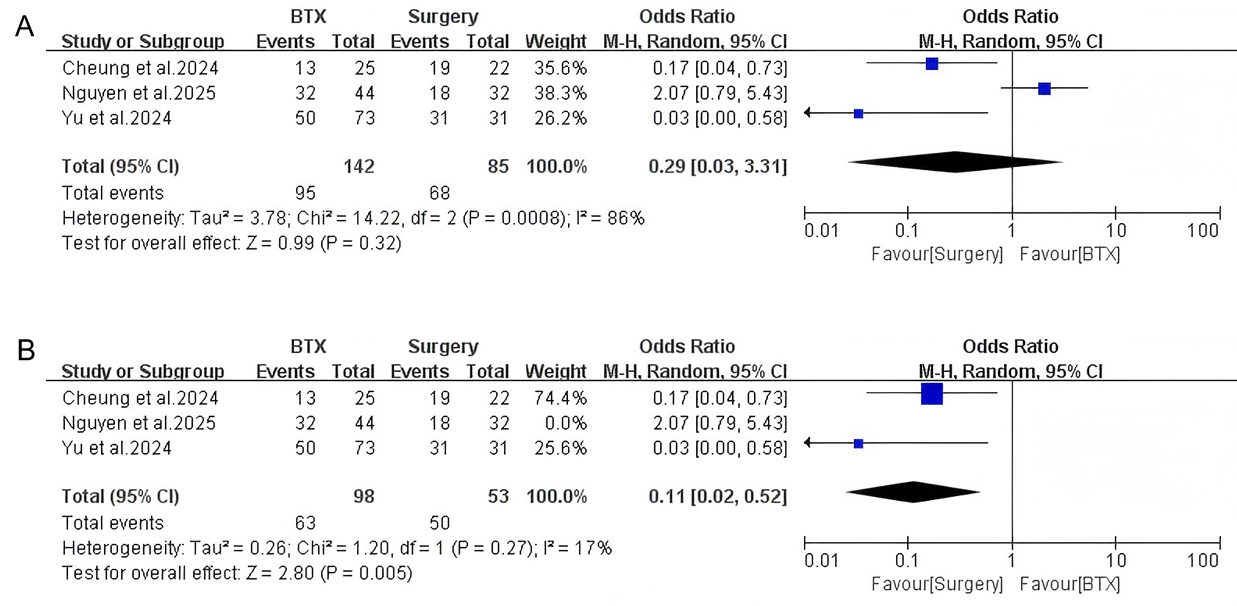


Figure S2. Forest plot comparing the motor success rate of BTX injection and surgery for AACE at 24-36 months. (A: Results of analysis before sensitivity analysis. B: Results after sensitivity analysis, in which the study by Nguyen et al. was excluded.) Given the limited number of studies, substantial heterogeneity, and instability observed in the sensitivity analysis, these findings should be interpreted with caution and are intended for exploratory purposes only.


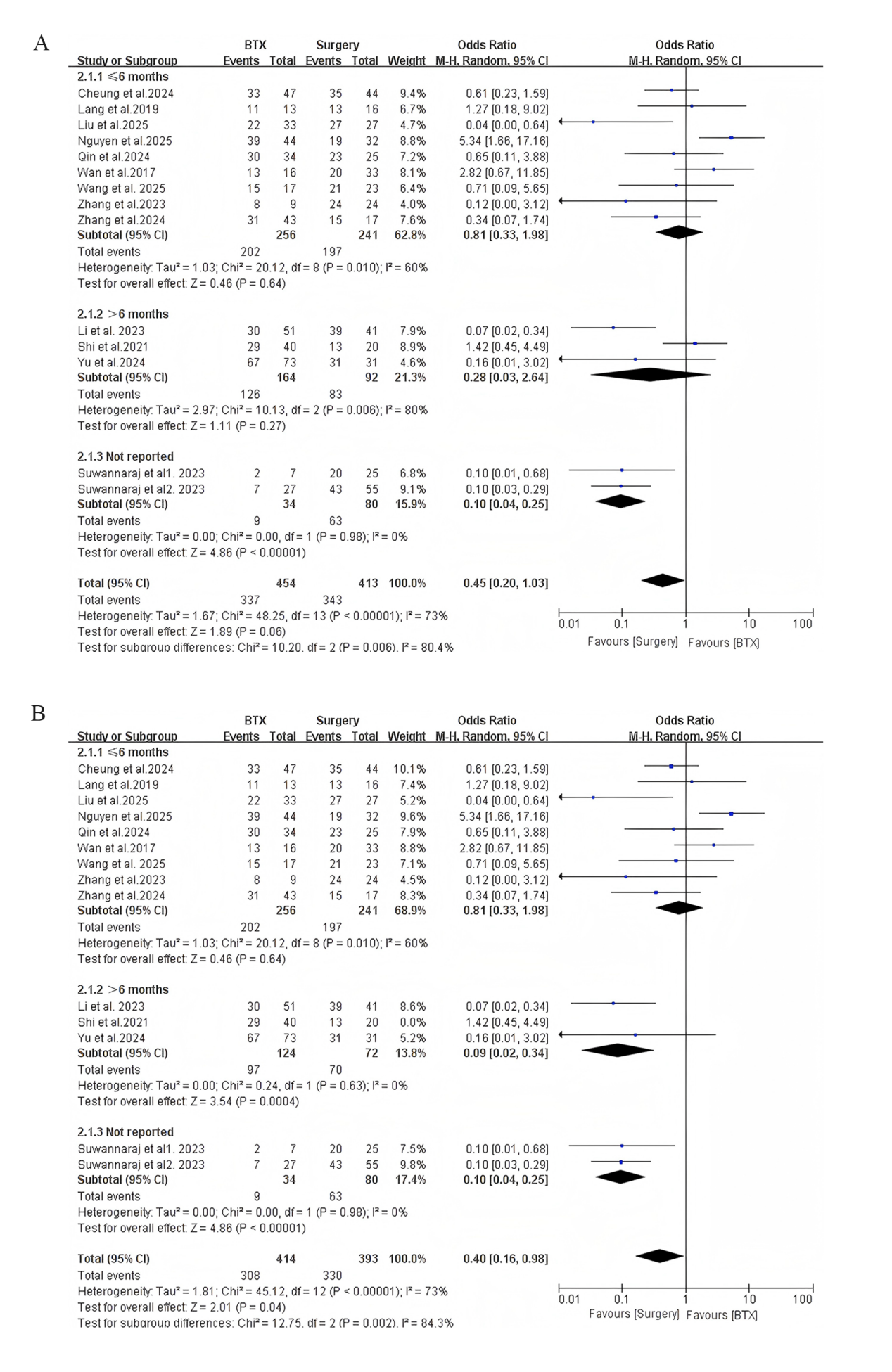


Figure S3. Forest plot of subgroup analysis based on AACE duration. (A: Results of subgroup analysis before sensitivity analysis. B: Results after sensitivity analysis, in which the study by Shi et al. was excluded from the subgroup with duration >6 months; results for the subgroup with duration ≤6 months remained unchanged.) Due to the limited number of studies, substantial heterogeneity, and instability in the subgroup with duration >6 months, the findings should be interpreted with caution and are presented for exploratory purposes only.


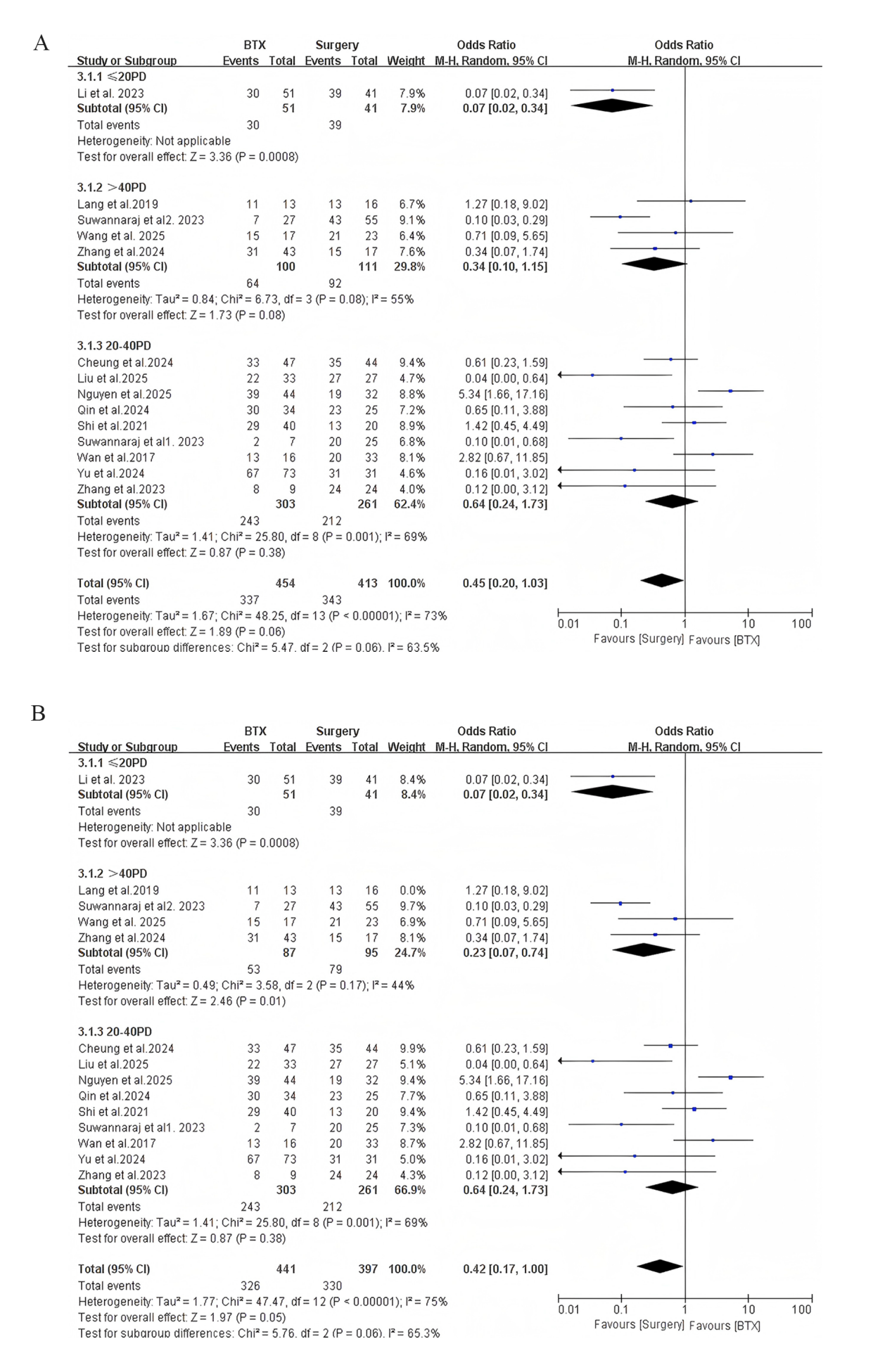


Figure S4. Forest plot of subgroup analysis based on preoperative mean deviation of in the BTX group. (A: Results of subgroup analysis before sensitivity analysis. B: Results after sensitivity analysis, in which the study by Lang et al. was excluded from the subgroup with preoperative mean deviation＞40PD; results for the 20-40PD subgroup remained unchanged.) Due to the limited number of studies and instability in the subgroup with preoperative mean deviation＞40PD, the findings should be interpreted with caution and are presented for exploratory purposes only.
